# Exploration of Bioactive Compounds, Antioxidant and Antibacterial Properties, and Their Potential Efficacy Against HT29 Cell Lines in *Dictyota bartayresiana*

**DOI:** 10.3390/md23060224

**Published:** 2025-05-23

**Authors:** Durairaj Swarna Bharathi, Andiyappan Boopathy Raja, Suganthi Nachimuthu, S. Thangavel, Karthik Kannan, Sengottaiyan Shanmugan, Vinaya Tari

**Affiliations:** 1 PG and Research Department of Zoology, Nehru Memorial College, Affiliated to Bharathidasan University, Puthanampatti 621004, Tamil Nadu, India; boopathyraja07@gmail.com; 2 PG and Research Department of Physics, Government Arts College (Autonomous), Affiliated to Bharathidasan University, Karur 639005, Tamil Nadu, India; suganthiphyupm@gmail.com; 3 Department of Physics, C.S.I. Bishop Solomon Doraisawmy College of Arts and Science, Karur 639001, Tamil Nadu, India; suhithangam@gmail.com; 4 Department of Mechanical Engineering, Advanced Institute of Manufacturing with High-Tech Innovations, National Chung Cheng University, Chia-Yi 621301, Taiwan; 5 Research Centre for Solar Energy, Integrated Research and Discovery, Department of Physics, Koneru Lakshmaiah Education Foundation, Green Fields, Vaddeswaram, Guntur 522502, Andhra Pradesh, India; shanmugan@kluniversity.in; 6 Department of Biology, Faculty of Science and Technology, Universitas Airlangga, Surabaya 60115, East Java, Indonesia

**Keywords:** *Dictyota barteyresiana*, HP-TLC analysis, GC-MS, antioxidant activity, antibacterial activity, HT 29 cancer cells

## Abstract

This study investigates the rare seaweed alga *Dictyota bartayresiana* lamour for biological activity. Antioxidant and antibacterial activities were examined. An MTT assay was carried out to examine cytotoxicity activity against colon cancer cells. The HPTLC analysis was performed for four different extracts, which exhibited clear flavonoid band formation at 254 nm and 366 nm with varied ranges of R_f_ values: methanolic extract (R_f_ 0.87), acetone extract (R_f_ 0.82), and benzene (R_f_ 0.83). Methanolic Extract Fraction One (MEF1) has a distinct band formation at 366 nm, it is shown to have the highest inhibition (6.20 ± 0.53 mm) against *Escherichia coli*, and the MTT assay reveals that the aqueous extract of *Dictyota bartayresiana* extract has an IC_50_ value of 300 µg/mL. It is divulged that methanolic extract shows the highest phytochemical compound level among the four extracts of *Dictyota bartayresiana*. A GC/MS analysis was employed to investigate the flavonoid profile of the crude seaweed extract. Although LC/MS is typically preferred for flavonoid analysis due to thermal sensitivity, GC/MS was used in this study owing to time constraints, with optimized conditions to reduce thermal degradation. The GC-MS analysis identified Quinoline and other flavonoids, suggesting potential bioactivity. The cytotoxicity activity of MEF1 shows that the development of a promising drug may be evaluated from a seaweed source. The present study provides excellent insight with the first report of the biologically active compound of *Dictyota bartayresiana*.

## 1. Introduction

Seaweed is a valuable source from the marine environment and can survive with plenty of struggles. According to Darwin’s postulation, the “Ability of fitness of organism progressively increased by adapting themselves for their corresponding environment with natural support” [1]. This competence may be biotic, or abiotic factors may encourage the development of physical or chemical changes in populations. External forces stimulate secondary metabolite production, which is tightly enveloped with the organism’s evoluation and defense against predators, which mainly involves the eradication of specific organisms such as fungi, bacteria, viruses, inflammatory substances, free radicals, etc. The secondary metabolites have strengthened species in various ways [2,3,4]. Ancestors knew that algae were used as a food supplement, and recent research has found that algae contain phytochemical compounds that can help to treat various diseases [5,6].

Seaweed possesses anticoagulant properties, preventing excessive blood clotting [7]. It produces a wide range of chemically active compounds in its environment, which show antibacterial, antifungal, antimacrofouling, and other pharmacological properties [8]. Compounds, like chlorophyll-a, fucoxanthin, and phenols, and flavonoids assist seaweeds in scavenging free radicals capably, suppress oxidation enzymes, and provide other favorable bioactive properties. Seaweeds containing both water and lipid-soluble vitamins, including thiamine and riboflavin, β-carotene, and tocopherols, which may lower the risk of heart disease, thrombosis, and atherosclerosis, neutralize free radicals and suppress the extent of oxidative deterioration [9]. The reactive oxygen species (ROS) formed in human tissues can promote extensive oxidative damage that leads to age-related degenerative processes, cancer, and a wide range of other human diseases [10]. An enormous variety of fibers is present in seaweed, especially fucose, mannose, galactose, and uronic acids, which depend on the seaweed group [11]. Seaweed fiber contains structural polysaccharides in brown seaweed (alginate and fucoidan), red seaweed (carageenan, agar, and porphyrin), and green seaweed (ulvan). A previous investigation reported that polysaccharides and oligosaccharides extracted from seaweeds may improve intestinal function, prevent pathogen adhesion and avoidance, and potentially cure inflammatory bowel disease. Anticoagulant, antitumor, and antihyperlipidemic effects are also exhibited in certain seaweed polysaccharides [12].

Seaweeds’ composition includes steroids, phenols, tannins, saponins, flavonoids, terpenoids, and glycosides, which have been extensively studied and used in therapeutic applications. Tannins have been found to exhibit antimicrobial properties, as they can bind to adhesins and are involved in enzyme inhibition, substrate deprivation, and membrane disruption [13]. Saponins have such specific biological activities as anticancer, anti-inflammatory, antimicrobial, and antioxidant properties [14]. Saponins also have the property of precipitating and coagulating red blood cells [15]. Flavonoids are hydroxylated phenolic constituents that are known for their response to antioxidant activity [16]. Steroids play a vital role in antimicrobial, anticancer, antiarthritic, antiasthma, and anti-inflammatory properties [17]. Terpenoids extensively show their cytotoxicity against a variety of cancer cells and cancer prophylaxis; on the other hand, glycosides can be used as food additives and bio-preservatives [18,19].

Seaweed prevents the growth of microorganisms that commonly cause diseases such as diarrhea, skin infections, infection in the internal organs, bloodstream infections, bone and joint infections, pneumonia, etc., in human beings and acts as an antimicrobial component [20,21]. It also cures the inflammation and rupturing of intestinal endothelial cells and acts as an anti-ulcer drug. It acts as an anti-inflammatory substance by repairing and treating tissue injuries [22]. Seaweed helps with its radiological activities in treating dreadful diseases like cancer, so it can be termed an anticancer agent [23]. It is used as an antiviral agent for humans infected with viruses [24,25]. In addition, it helps in the scavenging of free radicals, which can be proven by in vitro studies, and it also acts as an antioxidant agent [26].

Seaweed has been broadly categorized as Chlorophyta, Phaeophyceae, and Rhodophyta based on its nutrient content and chemical composition [27]. Taxonomical studies have revealed that seaweeds deviate from terrestrial plants by their absence of vascular tissue, roots, shoots, and flowers. The chlorophyta community is commonly found in nature. There are more than 7000 species present in a range of ecological systems like freshwater, marine, and terrestrial habitats [28]. Among the three phyla, only two phyla are considered to have predominant levels of applications in industry as well as in food consumption, Phaeophyceae and Rhodophyta. Phaeophyceae, unlike Chlorophyta, dominate marine environments, and less than 1% of them reside in freshwater ecosystems. They contain chloroplasts, which are surrounded by four membranes of three thylakoid stacks and a polysaccharide membrane. The polysaccharide membrane acts as a potential outer covering that elicits the biological activities of organisms and protects them from external environmental struggles [29]. Rhodophyta differ from other phyla by three important characteristics: thylakoids without stacks, external endoplasmic reticulum unseen in chloroplasts, and absence of flagella, but they possess two major accessory pigments, known as phycoerythrin and phycocyanin. Of these phyla, dominant in marine environments, only 3% (5000 species) thrive in freshwater habitats [30].

They largely consist of minerals, minor levels of carbohydrates, proteins, carotenoids, vitamins, and essential fatty acids; other than agar-agar, alginate and carrageenan can also be extracted from seaweed. In coastal areas, people are consuming seaweed as an important dietary source because of its potential nutritional content and its delicious nature, especially in Asian countries, which largely export seaweed to various countries for medicinal purposes and foodstuffs. Medicinal fields have utilized seaweed as a valuable component [31]. This paper describes *Dictyota bartayresiana*, which belongs to the brown algae family, and the external structure indicates that homogenized segments are less than four breadth long, but internodes are longer; the upper sinuses are broad and the sinuses are usually narrower.

This study gives a new insight into a few reports about *Dictyota bartayresiana* on its total bioactive contents and biological activities with mass spectroscopic analysis. To supplement the current investigation with a new potential source of cytotoxicity properties, the antibacterial properties and the antioxidant activities of extracts against a range of radicals (ABTS, nitric oxide, and hydrogen peroxide radicals) were examined, as well as their phytochemical contents (total protein, total flavonoid, total tannin, total carbohydrate, total ash, and total fat), and a GC-MS analysis was performed.

## 2. Results and Discussion

### 2.1. Phytochemicals Screening

The phytochemical screening was performed through various tests for the occurrence of resins, glycosides, saponins, steroids, tannins, terpenoids, flavonoids, phenols, alkaloids, and carbohydrates, as well as the absence of notable compound gums, as illustrated in Table 1 [32,33].

### 2.2. Quantitative Analysis (Determination of Compounds)

The dried *Dictyota bartayresiana* was estimated to contain a quantity of phytochemical compounds, such as flavonoids and tannins. The flavonoids showed 0.92%, and the tannins showed 0.09%. The primary compounds of ash, fat, and carbohydrates were also estimated; the total for ash was 37.4% higher than the protein, which contained 20.8%, the carbohydrates exhibited 8.43%, and the fat contained 2.25% (Table 2).

### 2.3. Thin-Layer Chromatography

TLC is the confirmation analysis for identifying the phytochemical compound in the extracts by forming unique bands in the TLC plate. There were four alcoholic extracts examined under a constant mobile phase, but the methanolic extract showed a dark yellow–brown band formation and appeared dark on green fluorescence UV light at 254 nm, rather than ethanol, acetone, and benzene. Among these, methanolic extracts were taken into consideration for the following analysis. The R_f_ value indicated the presence of a flavonoid compound in the methanolic extracts of *Dictyota bartayresiana*.

### 2.4. Column Chromatography

The current analysis was started to find out the presence of flavonoid compounds in the selected seaweed, which was confirmed by column chromatography. Using column chromatography, a flavonoid was specifically purified from seaweed. The purified form of the flavonoid was confirmed once again by a preliminary analysis, i.e., phytochemical screening.

### 2.5. GC-MS Evaluation

**Figure 1:**
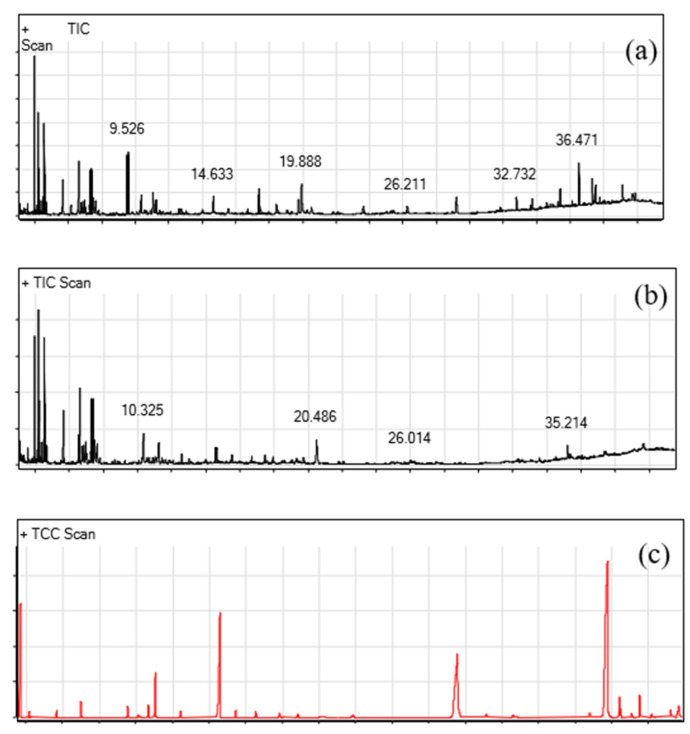
GC-MS interpretation. (**a**) GC-MS analysis of peak variation for *D. bartayresiana* crude extract. (**b**) GC-MS analysis of peak variation for *D. bartayresiana* extract fraction I. (**c**) GC-MS analysis of peak variation for *D. bartayresiana* extract fraction 2.

The bioactive compounds present in two different fractions of the methanolic extracts and crude extracts, which were obtained from the *D. bartayresiana* seaweed, are shown in Tables S1–S3. Their identification and characterization were based on their elution order in a TLC scanner. The acquisition time, molecular formula, and biological activities are represented in the table. The chromatogram peaks were unified and related through the database of spectra of recognized components (Wily-275) stored in the GC–MS library. The described presentations of the GC–MS investigation of the extracts are listed in Tables S1–S3 (given in the Supplementary Information), and the peak variation of the *D. barteyresiana* crude extract is displayed in Figure 1. It is concluded that the presence of Hexadecane, Quinoline, and 1,2-dihydro-2,2,4-trimethyl compounds has received great attention due to the significant biological activity noted in previous reports [34,35,36], such as antimicrobial and cytotoxicity activity.

While LC/MS is commonly used for flavonoid analysis due to the prevention of thermal degradation of flavonoids, GC/MS was employed in this study due to time and equipment availability. The temperature program and analysis duration were optimized to minimize thermal decomposition, and compound identification was supported by comparison with authentic standards and NIST library spectra.

### 2.6. HP-TLC Profile

**Figure 2:**
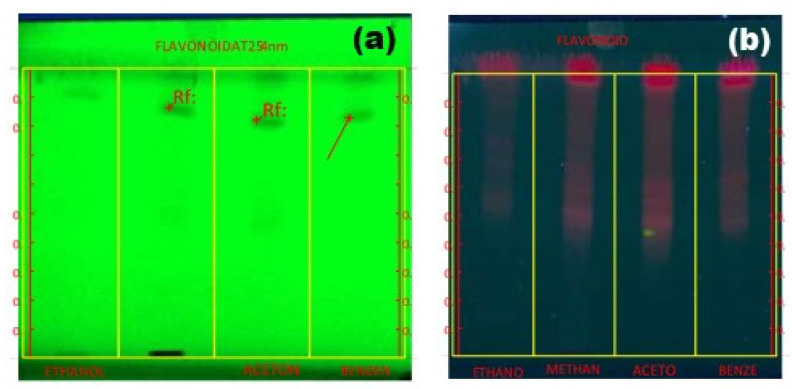
(**a**,**b**) HP-TLC results for *Dictyota bartayresiana* extract at two different nanometers.

High-performance thin-layer chromatography (HP-TLC) was used for identifying phytochemical compounds in seaweed extractions with highly qualified photographic figures, which are responsible for improving the medicinal value of seaweed. The identification of the bioactive compounds can be carried out from selected seaweed, extracted by using four different solvents, and their Rf values can be compared with known standards. The flavonoid compound appeared in the light red and dark red fluorescent bands at 366 nm in the extractions of ethanol, methanol, acetone, and benzene, whereas at 254 nm, the compound appeared in the slight brown band and dark brown bands in each extraction. The R_f_ values are given below 1.00, 0.57, 0.01, 0.13, 0.53, 0.61, 0.65, −0.01, 0.68, 0.78, and 0.74. From these Rf values, those of 0.13 and 0.61 represent the presence of myricetin and galangin, respectively, based on the standard R_f_ value (Figure 2 and Table 3). The presence of flavonoid compounds in Figure 2a was measured at 254 nm, which depicts the light band lane when compared to 366 nm. Among four different extracts, from right to left, ethanol, methanol, acetone, and benzene, the methanol showed a dark lane and point. Myricetin is a common plant-derived flavonoid and is well recognized for its nutraceutical value. It is one of the key ingredients in various foods and beverages. The compound exhibits a wide range of activities, which include strong anti-oxidant, cytotoxicity, antidiabetic, and anti-inflammatory activities. Previous studies have reported that galangin and myricetin inhibited cell growth in several cancer cells, such as hepatoma, pancreatic, esophageal, melanoma, gastric, and colon carcinoma cells [37].

**Figure 3:**
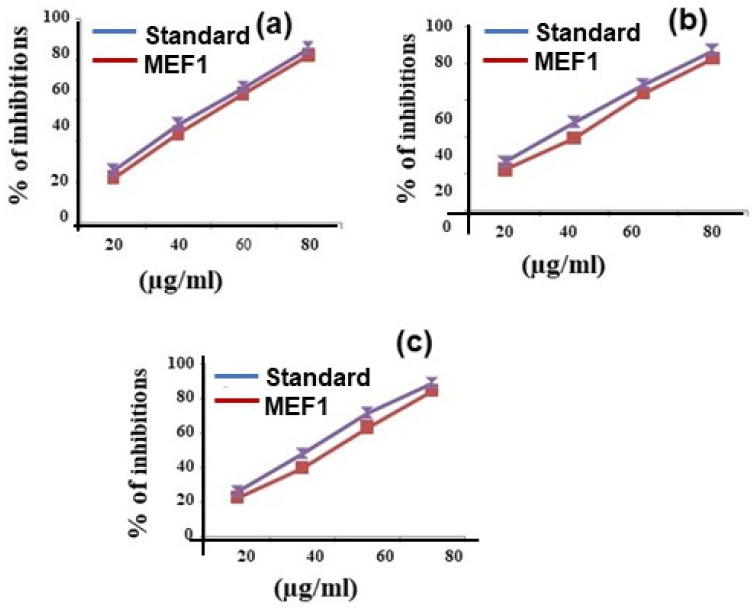
Antioxidant activity of Methanolic Extract Fraction One for (**a**) nitric oxide, (**b**) hydrogen peroxide, and (**c**) ABTS radical scavenging assay.

Figure 3 reveals the antioxidant activities of the free radical scavenging capacity of synthesized samples in different dosages in addition to standard drugs.

The antioxidant parameters were as follows: nitric oxide scavenging activity, hydrogen peroxide decomposition assay, and ABTS assay. These values are duly obtained from the half inhibition rate of the IC_50_ values (Table 4). In the hydrogen peroxide decomposition activity, the ability of the extract to decompose hydrogen peroxide was assessed, which may involve both the direct action of antioxidant compounds and the possible influence of trace metals present in the extract through the Fenton reaction. The presence of metals can catalyze the degradation of hydrogen peroxide and, therefore, the effect observed may not solely be due to the compounds in the extract. To accurately evaluate the contribution of the compounds, future experiments could include the use of chelating agents to minimize the influence of metal ions.

The scavenging activities fluctuated according to the concentration used in the analysis; 80 (µg/mL) exhibited the best inhibition percentage, which decreased following dosages of 60 (µg/mL), 40 (µg/mL), and 20 (µg/mL). All the parameters merely had similar inhibition values (µg/mL); the NOS, HPS, and ABTS assay IC_50_ values were 47.91, 47.61, and 46.89, respectively.

**Figure 4:**
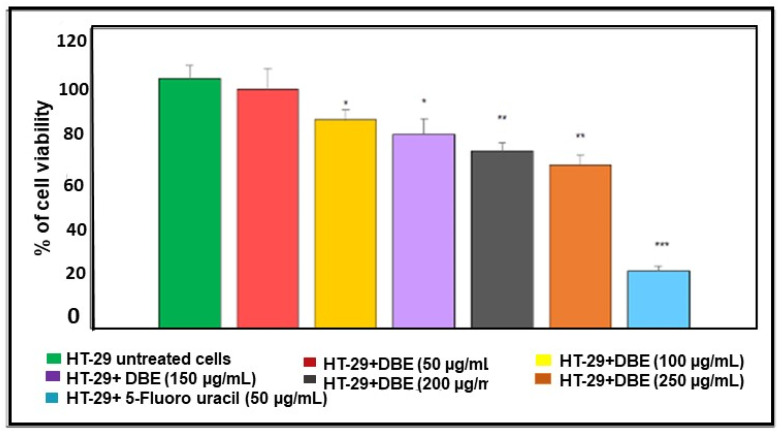
HT-29 cell viability percentage in the treated and untreated groups. Values are stated as Mean ± SEM (n = 3); * *p* < 0.05; ** *p* < 0.01; *** *p* < 0.001; statistical significance as evaluated with HT- 29 untreated.

The MEF1 of *Dictyota bartayresiana* was tested for cytotoxicity activity in the colon cancer cell line HT 29. The samples were analyzed for anti-carcinogenic action in different concentrations of 50, 100, 150, 200, and 250 µg/mL. The sample showed concentration-dependent cytotoxicity activity of the HT29 colon cancer cell line. It showed an inhibition concentration in terms of an IC_50_ value of 300 µg/mL. The seaweed extract can slow down the growth of HT29 cell lines as small as 50 µg/mL, which is depicted in Table 5 and Figure 4. *Dictyota bartayresiana* has cytotoxicity properties against colon cancer cells because of the existence of beneficial bioactive compounds. Molecular docking studies of *Dictyota bartayresiana* have proven their capacity to invade colon cancer cells and provide potential support for current research.

The MEF1 extract has proven its biological activities through the following assays: nitric oxide scavenging assay, hydrogen peroxide ABTS assay, MTT assay, and well diffusion assay. Among all these assays, the MTT assay shows significant value against colon cancer cell lines. The presence of flavonoids and other phytochemical compounds in the MEFI extract may be the major reason for its cytotoxicity.

Many reports support the significant anticancer action of phytochemical compounds [38,39]. The flavonoid could interfere with cancer cell line arrest of the cancer cycle at the G_2_ phase, which promotes DNA damage. It can inhibit thymidine kinase, which is responsible for further growth of the cell. The tumor suppression gene plays a vital role in the prevention of cancer; when it is activated by a stimulus, cell growth can be stopped. Flavonoids can activate the tumor suppressor gene to reduce the further development of cancer cells. Apoptosis (or) cell death is natural when cells decide to die by themselves. This process could be a targeted step for all kinds of anticancer drugs. Flavonoids can provoke the apoptosis mechanism and allow cancer cells to die, and cease further development [40,41,42,43,44,45,46]. The standard anticancer drug, 5-fluorouracil, also involves the above events. Fortunately, flavonoids of phytochemical compounds have been studied in cancer research [47]. Numerous mechanisms still need to be investigated further. Oncogenesis is a broad spectrum when it deals with phytochemical compounds and could be a side-effect-free, easily available, large-scale production in the future.

**Figure 5:**
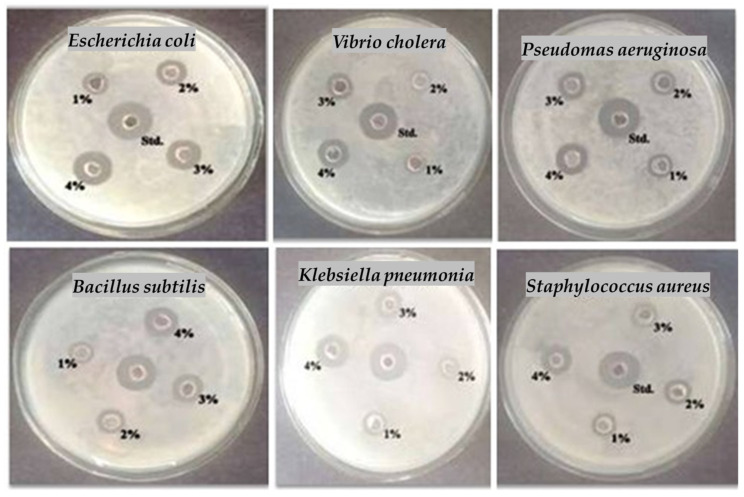
Antibacterial activities of the methanolic extract of fraction 1 for Gram-positive and Gram-negative bacteria.

The current investigation suggests that among eighteen bacteria, six bacteria have shown the best effects, as presented in Figure 5. The MEF1 antibacterial activity was examined against *Escherichia coli*, *Vibrio cholerae*, *Pseudomonas aeruginosa*, *Bacillus subtilis*, *Klebsiella pneumonia*, and *Staphylococcus aureus* (Table 6). The concentration of the sample is directly proportional to the inhibition percentage. The maximum action of *Escherichia coli* (6.20 ± 0.53 mm) was detected in a 4% dilution of MEF1. The minimum activity (0.60 ± 0.04) for *Vibrio cholerae* was obtained in a 1% dilution of MEF1, and it has possible activity in every concentration, and adjacent to the standard dose, as shown in Figure 5.

Herbal medicines are commonly used as drugs for many lifesaving treatments, especially in the Siddha and Ayurvedic methods of medicine. Ancestors knew plenty of terrestrial plants and explained their morphological, anatomical, as well as medicinal value. Their editions were helpful for upcoming generations, but their knowledge of marine organisms had few countable records due to their marine origin, and they were focused only on their visible nature and failed to make observations about the behind-the-scenes of the marine world. Currently, researchers are curious about marine organisms that can survive in several problematic environments with the help of secondary metabolites. They have discovered that a bioactive compound may be easily extracted from marine algae. In the current investigation, we evaluated the bioactive compounds in the *Dictyota bartayresiana* marine alga by the GC-MS method. 

The primary constituents were estimated in seaweed extract according to the standard methods, and the highest content of the sample was ash (37.4%), followed by protein (20.8%), carbohydrate (8.43%), and total fat (2.25%). Table 1 and Table 2 of the quantitative analysis of the phytochemical compounds results is considered as supporting evidence for the flavonoid group (0.92%) in the extract, rather than tannins (0.09%).

The crude extract contains Dihydroxyphenyl (C_10_H_12_O), Methoxyacetopheone (C_9_H_10_O_2_), Naphthalene (C_10_H_8_), Desmethyl selegiline (C_12_H_15_N), Zingerone (C_11_H_14_O_3_), Quinoline (C_12_H_15_N), Methyl palmitate (C_17_H_34_O_2_), Behenic acid (C_22_H_44_O_2_), and Hexadecane (C_16_H_34_). Every compound shows a pharmacological function of its own. For instance, the Dihydroxyphenyl compound, otherwise known as coniferyl alcohol, exhibits good antidiabetic activity, and this compound is present in the queen bee and important constituents of the pheromone group, which involves the retinue pathway. Methoxyacetopheone acts as a food additive in the USA [48], Zingerone and Quinoline are considered as a novel drug that promotes antioxidant and antibacterial activity [34], and Desmethyl selegeline is a potential compound in opposition to N-methyl-D-aspartate-induced rat retinal damage [35]. Hexadecane is a potential antifungal agent whose activity is elicited due to its volatile constituents [36].

Fraction 1 contains Fisetin (C_8_H_24_O_4_Si_4_), Zingiberene (C_15_H_24_), Dihydromyristicin (C_11_H_14_O_3_), Hexadecane (C_16_H_34_), Diethylhexylphthalate (C_24_H_38_O_4_), and Hexadecane (C_16_H_34_) components. Fisetin has potential anticancer activity against cancer cells, especially in ovarian cancer [49]. Myristicin is a successful inhibitor of B[a]P-induced tumor formation. This type of disease is commonly exposed to the environment by polycyclic aromatic hydrocarbon [50], which is a breakdown product of Dihydromyristicin. Acinetobacter in bacterial strains could make use of Hexadecane and phenolics considered as carbon and energy sources for their growth [51]. DHP’s actions are different from species to species, and these actions suppress the growth of tumor cells in rats, promote hepatocyte growth regulation, and induce DNA synthesis [52].

Fraction 2 contains a few phytochemical compounds, such as Isopropenylphenol (C_9_H_10_O), Quinoline, 1,2-dihydro-2,2,4-trimethyl-(C_12_H_15_N), Naphthalene (C_10_H_8_), n-Tridecan-1-ol (C_13_H_28_O), and n-pentadecanol (C_15_H_32_O). Quinoline is an essential constituent, which has an electron-donating amino group that promotes the free radical scavenger action of components, and a vital oil of n-pentadecanol has the property of acting against bacterial pathogens [53].

There is no available research on the total flavonoid, tannin, total ash, carbohydrate, protein, and total fat content in *D. bartayresiana.* Commonly, the antioxidant action of plants may be directly contributed by phenolic compounds. Tannins and flavonoids are well-known medicinal agents that possess antioxidant and anti-inflammatory properties [54,55,56]. The high-performance thin-layer chromatography technique was performed for obtaining the best photographic figures for analyzing the flavonoid wavelengths of various extracts, and the application positions changed depending on the solvents (Table 3). In previous work, we carried out molecular docking to analyze the binding properties of the mediator called 3HNG with Hexadecane, hexadecanoic acid methyl ester, and Quinoline,1,2-dihydro-2,2,4-trimethyl) reported from *Dictyota bartayresiana*, and 5-β fluorouracil used as a standard. The wet analysis carried out by us showed the best result with 3HNG and proved the presence of an anti-colon cancer property. Among the various compounds, hexadecanoic acid methyl ester and Quinoline, 1,2-dihydro-2,2,4-trimethyl) had higher binding energy than the standard. The present study may strongly conclude that the seaweed extract displays cytotoxicity against colon cancer cells [47].

## 3. Methodology

### 3.1. Preparation of Extract and Identification

The seaweed was collected from the Mandabam area in November 2018, and authenticated by the Botanical Survey of India (BSI), Coimbatore, India. A voucher sample has been placed in the herbarium of BSI. Reg no: BSI/SRC/5/23/2019/Tech/3070). Seaweeds that had been shade-dried were ground into powder and dissolved in acetone, methanol, ethanol, and benzene (10 g of plant sample in 100 mL solvent). For 6 to 8 h, the solvent extracts were put through the Soxhlet apparatus [57], depending on the phytochemical split-up.

### 3.2. Screening of Phytochemical Compounds

**Figure 6:**
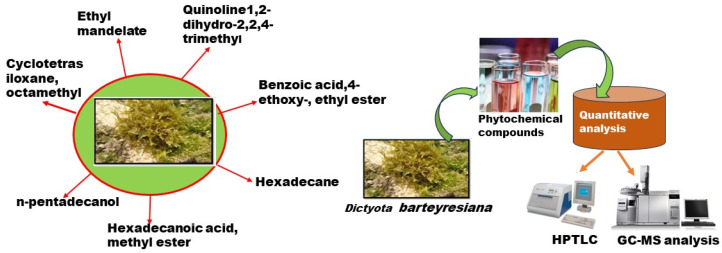
Detection of valuable compounds from *Dictyota bartayresiana* extract.

The phytochemical compounds were screened out through standard procedures, which indicate the presence or absence of bioactive compounds in a crude extract. Figure 6 shows the schematic presentation of the detection of compounds from *Dictyota bartayresiana* extract.

### 3.3. Quantitative Analysis

#### 3.3.1. Estimation of Protein

Seaweed powder (100 mg) was homogenized with 3 mL of 10% trichloroacetic acid. The samples were centrifuged at 10,000 rpm. Supernatants were removed. In total, 3 mL of 1 N sodium hydroxide (NaOH) pellets were added to each sample, and samples were allowed to cool for seven minutes in a water bath. The samples were again centrifuged for 5 to 10 min at 5000 rpm. After centrifugation, 5 mL of reagent having 0.5 mL of supernatant was mixed with 100 parts of a 2% sodium carbonate mixture and one part of a 2% sodium potassium tartrate mixture. The supernatant was kept for 10–15 min. Finally, the supernatant was added to 5 mL of Folin–Ciocalteu’s phenol reagent and left undisturbed for half an hour to examine the progress of color, and the absorbance was also measured at 700 nm [58].

#### 3.3.2. Estimation of Flavonoids

Total flavonoid contents were estimated by using the aluminum chloride approach, in which quercetin was used as a standard. The seaweed excerpt was adjoined with distilled water and 5% NaNO_2_ was added drop by drop. This sample was kept at 25 °C for 5 min, and then AlCl_3_ was added. These mixture reagents were kept to cool for 5 min, with 0.2 mL of 1 mM NaOH added. Finally, 1 mL of water was added to the reaction mixture. The absorbance was measured at 510 nm [59].

#### 3.3.3. Estimation of Tannins

The seaweed crude extract was diluted using 50 mL of water and maintained in a water bath through continuous stirring for 30 min. The supernatants were transferred into a volumetric flask, and the extraction process was overfed until the mixture became colorless. The resulting solution was cooled and then mixed with 25 mL indigo sulphonic acid solution. The resulting content was titrated by 0.1 M potassium permanganate solution with continuous stirring until the development of a yellow color. Each 1 mL of 0.1 M aqueous potassium permanganate is comparable to 0.004157 g of tannins. The total tannin content was determined depending on the titration value [58].

#### 3.3.4. Estimation of Total Ash

A total of 3 g of seaweed powder was taken in a silica crucible. It was incinerated quietly at first and the temperature was slowly raised to 475 °C ± 25 °C until a carbon-free state was cooled and weighed. The extract’s burned mass, including boiling water, the insoluble residuum was gathered over ashless filter paper, and the remains were burned and filtered until the ash was white or close; the filtrate was then added, dried to dryness, and then ignited and evaporated to dry out and the whole sample was heated to a temperature of 475 °C ± 25 °C.

The percentage of total ash is calculated as follows:

(1) $$\%\;of\;total\;ash\left(\frac{\%w}{w}\right)=\frac{Weight\;of\;ash\left(g\right)\times 100}{weight\;of\;the\;sample\;(g)}$$

#### 3.3.5. Estimation of Total Fat

A total of 3 g of seaweed powder was taken into the Soxhlet Apparatus. The Soxhlet apparatus condenser and solvent flask were connected. The solvent was added in the required volume, and the boiling point varied between 40 and 60 °C. The extract was continuously monitored until the colorless solvent was obtained, which took 16 h. The thimble was removed, and the solvent was evaporated and weighed [58]. The flask was cooled and weighed (‘b’,g).

(2) $$\%\;Fat\;Content\left(\frac{\%w}{w}\right)=\frac{\left(b-a\right)\left(g\right)\times 100}{Weight\;of\;the\;sample\left(g\right)}$$

#### 3.3.6. Estimation of Total Carbohydrates

A total of 100 mg seaweed powder was added, with 5 mL of 2.5 N HCl taken in a hot tube, kept in water for 3 h, and then cooled to the optimum temperature. A neutralization state was obtained by adding sodium carbonate to the reaction mixture. The resulting mixtures were centrifuged, and the supernatant was collected. Glucose standard mixtures were arranged into 0, 0.2, 0.4, 0.6, 0.8, and 1 mL of the running standard. In total, 4 mL of anthrone reagent was added to the sample solution. The volume was made up of 5 mL of each tube. The sample solution was kept in a hot water bath for 8 min. Absorbance was examined at 630 nm [60].

(3) $$\%\;Total\;Carbohydrates=\frac{Sample\;absorbance}{Standard\;absorbance}\times \frac{Standard\;weigth}{Standard\;dilution}\times \frac{Sample\;dilution\times 100}{Sample\;weight}$$

### 3.4. Thin-Layer Chromatography

TLC was used to analyze the bioactive compounds in the extracts with the help of R_f_ values. In this technique, silica gel precoated plates were used, and a ratio of ethyl acetate (100): formic acid (11): glacial acetic acid (11): water (26) of the mobile phase solvent system was utilized for determining the phytochemical compound. The extracts were placed on a silica plate in a drop-by-drop manner, which took place above the mobile phase system. Every bioactive compound has a particular polarity [57,61,62]. After a few minutes, active compounds were separated according to polarity. The plates were kept for air drying and sprayed with an iodine vapor background to examine the flavonoid compound [63].

R_f_ value can be expressed by

(4) $$R_{f}=\frac{Distance\;moved\;by\;solute}{Distance\;moved\;by\;solvant}$$

### 3.5. Refinement of Active Compounds

The chromatography technique was used to separate the desired compound from the bulk of the compound in the extract. Based on the standard procedure, silica gel was used as a static phase along with a chloroform (5): ethyl acetate (4): formic acid (1) solvent system, followed by chloroform (15): methanol (1) as nonpolar solvents, which helped to separate the flavonoid compound into individual fractions. From this, column value −30 cm in analytical scale, standard phase–silica gel, mobile phase—ethyl acetate: formic acid: glacial acetic acid: water. The continuous separation was performed using the appropriate solvent system in different ratios. There were twelve different fractions obtained from this analysis. After confirmation by TLC, two fractions were subjected to Gas–Mass Spectroscopy [64].

### 3.6. Gas Chromatography–Mass Spectroscopy

GC–MS investigation was performed using an Agilent Technologies system following previously reported methods [57,61,62], with the TNTH_SCAN_45Min_300 °C.M method and an autosampler. Gas chromatography was carried out using a fused silica capillary column (30 m × 0.25 mm i.d., 0.25 µm film thickness). Helium gas was used as the carrier at a flow rate of 1.51 mL/min for 1 min. The mass spectrometer operated in electron impact (EI) mode at 70 eV, scanning a mass range of *m*/*z* 91–283. The split ratio was set to 1:10, with an injection volume of 1 μL. The column temperature program ensured a total run time of 37 min.

Compound identification in the crude seaweed extract was carried out based on peak evaluation, retention time comparison with authentic standards, and matching of the acquired mass spectra with those in the NIST mass spectral library [65]. Selected compounds were verified by injecting commercially available standard substances under identical GC–MS conditions to confirm both retention time and fragmentation patterns. This dual approach enhanced the reliability of compound identification and ensured accurate characterization of the extract components.

### 3.7. Preparative HPTLC

Preparative HPTLC was performed on silica gel 60F 254 by using a CAMAG TLC Scanner (CAMAG, Muttenz, Switzerland) with the development of a twin trough chamber of about 20 × 10 cm. The plant material was dissolved in the following solvents: ethanol, acetone, benzene, and methanol. Temperatures were maintained at 60 °C, and the mobile phase was prepared by chloroform (8.5): methanol (1): formic acid (0.5). The different solvent extractions were taken to compare bioactivity among them to find out the best solvent for further analyses. The extracts were subjected to the mobile phase solvent, which showed 4 different tracks, with an application position of 8.0 mm. Bioactive compounds were formed as a band with the help of an inert gas. The developed bands were allowed to dry, and photography was performed using TLC Scanner with Win CATS Planner software (1.4.10). UV light facilitated the visualization from two different wavelengths under 254 nm and 366 nm, respectively.

### 3.8. Preparation of Sample

The samples were taken in various concentrations to investigate in vitro antioxidant activity [66], which followed 20, 40, 60, and 80 µg/mL.

#### 3.8.1. Nitric Oxide Scavenging Activity Analysis

Nitric oxide radical scavenging activity was estimated, consistent with the previous method [67].

##### Procedure

According to the Griess-Ilosvay reaction, the formation of sodium nitroprusside in an aqueous mixture happens by the liberation of nitrite ions. The sample of 0.5 mL was taken in a prepared reaction mixture of 0.5 mL phosphate buffered saline and 2 mL of 10 mM sodium nitroprusside at a mixture of concentrations, and the sample solution was set aside at 25 °C for 150 min. Subsequently, a 0.5 mL sample mixture was added to the 1 mL sulfanilic acid at room temperature for 5 min. Later, 1 mL of naphthyl ethylenediamine dihydrochloride was poured at room temperature, proceeding for 30 min. Absorbance was measured at 540 nm by a spectrophotometer. The nitric oxide inhibition action was resolved through the following calculation:

(5) $$\%\;Inhibition=\frac{\left(A_{0}-A_{1}\right)}{A_{0}\times 100}$$

where A_0_—absorbance of the control, blank, without sample, and A_1_—absorbance for the tested sample.

#### 3.8.2. Hydrogen Peroxide Decomposition Activity

The hydrogen peroxide decomposition activity of the sample was determined using the standard technique [68]. A total of 1 mL of extract was added in different doses of 1 mL of 0.1 mM H_2_O_2_, which was followed by adding 3% ammonium molybdate in the range of two drops, 7.0 mL of 1.8 M potassium iodide, and 10 mL of 2 M sulfuric acid. The reaction mixtures were titrated with 5.09 mM sodium thiosulfate, awaiting the fading of the yellow color. The percentage inhibition of hydrogen peroxide was computed as

(6) $$\%\;Inhibition=\frac{\left(V_{0}-V_{1}\right)}{V_{0}\times 100}$$

Titrated solution: sodium thiosulfate (NaS_2_O_3_) used for controlling the sample and solution volume represented as V_0_ in the presence of hydrogen peroxide (without sample), the volume of sodium thiosulfate solution represented as V_1_.

#### 3.8.3. ABTS Scavenging Assay

The prepared sample was determined for antioxidant effect employing ABTS decolorization assay from the previous method [69].

The ABTS radical cation formation was performed through the reaction of ABTS solution (7 mM) with 2.45 mM K_2_S_2_O_8_ (potassium persulfate). The sample solutions were undisturbed for 12 to 16 h in the dark at room temperature, which yielded the formation of (ABTS^•+^) radicals, as well as the appearance of dark color. The 1 mL ABTS reagent was dropped in different dilutions of the sample. After 6 min, the calorimetric absorbance was determined near 734 nm, which was evaluated with L ascorbic acid standard solution. Blank was utilized in each assay. The above antioxidant parameters were performed by triplicate, and the percentage inhibition was calculated via the following method:

(7) $$Inhibition\;(\%)=\frac{\left(Control-Test\right)}{Control}$$

### 3.9. Antibacterial Activities

Nutrient agar was added to the flask, accompanied by distilled water, which was also heated. The boiling agents are directly dispensed into a culture dish before the formation of the solidification process [70,71,72]. The diverse bacterial strains are injected into the culture vessels. For example, the following strains are injected into the culture plate: *Klebsiella pneumoniae*, *Escherichia coli*, *Enterobacter faecalis*, and *Flavobacterium* sp. *Klebsiella oxytoca*, *Proteus mirabilis*, *Providentiarettigeri*, *Salmonella typhi*, *Salmonella paratyphi*, *Serratia marcescens*, *Shigella flexineri*, *Shigella sonnei*, *Staphylococcus aureus*, *Methicillin-resistant S. aureus*, *Staphylococcus epidermidis*, *Vibrio cholera*, and *pseudomonas aeruginosa*. The agar dish was kept in an incubator at 37 °C. Chloramphenicol (30 µL) was used as a standard drug and positive control. The methanolic extract fraction was taken as a sample. Labeling of concentration facilitated obtaining the apparent results. The inhibition zone was assessed after 24 h. The antibacterial activity was achieved by a well-cut drug diffusion assay [70].

### 3.10. Cytotoxicity Study by MTT Assay

Human colon cancer cell adenocarcinoma (HT-29 cell line) was accessed from the National Centre for Cell Sciences (NCCS), Pune, India. To evaluate the cytotoxicity result of the methanolic fraction of the one extract of *Dictyota bartayresiana*, MTT assay was used. The colorimetric assessment was performed in 96-well plates. The whole experimentation was performed in sterilized states, and the sub-culturing technique was implemented according to the typical methods. The cell viability percentage was the result of the test drugs’ effect on cell growth inhibition, which was compared with the standard drug 5-fluorouracil (50 µg/mL). The experimental condition of 5-fluorouracil was considered a positive control.

### 3.11. Statistical Study

All test samples were studied for three individual factors. The quantity of the sample is mandatory to determine the free radical scavenging concentration through 50%, IC_50_, and was graphically further defined by linear regression method through MS Windows GraphPad Instat (version 3) software. Outcomes were extracted graphically/by mean ± standard deviation.

## 4. Conclusions

The above findings confirm the presence of several bioactive compounds in the extract. Among the identified constituents from *Dictyota bartayresiana* Fraction 1, long-chain fatty alcohols such as n-pentadecanol and n-nonadecanol, as well as isoflavones, were detected. These compounds are known for their notable antibacterial, antioxidant, and cytotoxic activities. Although these fatty alcohols are not classified as flavonoids, they may still play a significant role in the biological activity of the seaweed extract. This study offers valuable insight into the chemical profile of the extract and supports its potential for further pharmacological investigation.

The antioxidant activity of MEF1 (NOS) was demonstrated by its ability to scavenge free radicals, with an IC_50_ value of 47.91 µg/mL. The GC–MS analysis confirmed the presence of compounds such as Hexadecane, Quinoline, and 1,2-dihydro-2,2,4-trimethyl derivatives, which exhibited significant antibacterial and antioxidant properties.

## Figures and Tables

**Table 1:** Phytochemical screening of *Dictyota bartayresiana*.

S. No.	Tests	Experimental Conditions	Results
1	Test for alkaloids (Wagner’s test)	Seaweed extract added with 2 mL of Wagner’s reagent	Reddish brown precipitates appeared
2	Test for glycosides (Fehling’s test)	25 mL of dilute H_2_SO_4_ was added to 5 mL of seaweed extract and boiled, cooled, and neutralized with 10% sodium hydroxide, and then 5 mL of Fehling solution A and B were added	Red precipitate appeared
3	Test for phenolic compounds (ferric chloride test)	Seaweed extract was dissolved in 5 mL of distilled water. A few drops of neutral 5% ferric chloride solution were added	Dark green color appeared
4	Test for steroids and Terpenoids (Salkowski’s test)	Seaweed extracts were treated with chloroform and filtered. The filtrates were treated with a few drops of concentrated H_2_SO_4_, shaken gently, and allowed to stand.	Golden yellow color indicates the presence.
5	Test for carbohydrates (Benedict’s test)	0.5 mL of filtrate, 0.5 mL of Benedict’s reagent were added. The mixture was kept in a boiling water bath for 2 min.	Reddish brown color appeared.
6	Test for resins	Solubility test: seaweed extract was dissolved in different solvents	The extract dissolves completely, which indicates the presence of resins
7	Test for saponin	Foam test: 5 mL of seaweed extract in a test tube. Shake vigorously for 30 s and let it stand for 10 min	Persistent foam for 10 min indicates the presence of saponins
8	Test for tannin	In total, 3 g of a seaweed powdered sample was boiled in 50 mL distilled water for 3 min on a hot plate. The mixture was filtered, a portion of the filtrate was diluted with sterile distilled water in a ratio of 1:4, and three drops of 10% ferric chloride solution were also added.	The appearance of a blue color indicates the presence of tannins
9	Test for flavonoids	Ferric chloride test: 2 mL of extract added with a few drops of FeCl_3_ solution	Formation of blackish green color indicates the presence of flavonoid

**Table 2:** Test results of phytochemical estimation.

S. No.	Parameters	Results
1	Protein	20.8% *w*/*w*
2	Flavonoids	0.92% *w*/*w*
3	Tannins	0.09% *w*/*w*
4	Total Ash	37.4% *w*/*w*
5	Total fat	2.25% *w*/*w*
6	Carbohydrates	8.43% *w*/*w*

**Table 3:** High-performance thin-layer chromatography wavelengths of various extracts.

S. No.	Position	Volume	Vial	Sample ID	Active
>1	15.0 mm	5.0 µL	1	ETHANOL	Yes
>2	38.3 mm	5.0 µL	2	METHANOL	Yes
>3	61.6 mm	5.0 µL	3	ACETONE	Yes
>4	84.9 mm	5.0 µL	4	BENZENE	Yes

**Table 4:** Antioxidant parameters of Methanolic Extract Fraction One tested by nitric oxide, hydrogen peroxide, and ABTS radical scavenging assay.

Samples	% of Inhibitions				IC_50_ Value (µg/mL)
	20 (µg/mL)	40 (µg/mL)	60 (µg/mL)	80 (µg/mL)	
**Methanolic Extract Fraction One (NOS)**	22.38 ± 1.56	39.52 ± 2.76	63.81 ± 4.46	82.85 ± 5.79	47.91
**Ascorbic acid (Std)**	26.66 ± 1.86	48.09 ± 3.36	68.57 ± 4.79	87.14 ± 6.09	42.45
**Methanolic Extract Fraction One (HPS)**	22.50 ± 1.57	39.64 ± 2.77	63.21 ± 4.42	84.64 ± 5.92	47.61
**Ascorbic acid (Std)**	25.71 ± 1.79	47.85 ± 3.34	71.42 ± 4.99	88.92 ± 6.22	42.04
**Methanolic Extract Fraction One (ABTS)**	22.22 ± 1.55	44.22 ± 3.09	63.55 ± 4.44	82.44 ± 5.77	46.89
**Ascorbic acid (Std)**	25.77 ± 1.80	48.44 ± 3.39	66.66 ± 4.66	85.77 ± 6.01	43.28

Values mentioned as mean ± SD for triplicates.

**Table 5:** Cell viability results of colon cancer HT-29 by untreated, treated with *Dictyota bartayresiana* Methanolic Extract Fraction One (MEF1), and standard drug.

S. No	Treatment	Conc (µg/mL)	Absorbance 570 nm
1.	HT-29 by untreated cells	-	0.517 ± 0.05
2.	DBE treated	50	0.495 ± 0.03
3.		100	0.431 ± 0.02 *
4.		150	0.401 ± 0.03 *
5.		200	0.367 ± 0.02 **
6.		250	0.301 ± 0.02 **
7.	5-Fluoro uracil treated	50	0.118 ± 0.01 ***

Values are mean ± SEM stated as (*n* = 3); * *p* < 0.05; ** *p* < 0.01; *** *p* < 0.001; statistical significance tested for HT-29 untreated cells. Methanolic Extract Fraction One: IC_50_ value of the extract is 300 µg/mL.

**Table 6:** Antibacterial activity of Methanolic Extract Fraction One for Gram-positive and Gram-negative bacteria.

Tested Bacteria	Dose (50 µL) Sample				Std. (30 µL)
	1%	2%	3%	4%	
***Escherichia coli***	2.00 ± 0.14	3.10 ± 0.21	4.60 ± 0.32	6.20 ± 0.43	7.60 ± 0.53
***Vibrio cholera***	0.60 ± 0.04	1.80 ± 0.12	2.90 ± 0.20	4.10 ± 0.28	6.40 ± 0.44
***Pseudomas aeruginosa***	1.00 ± 0.07	2.20 ± 0.15	3.60 ± 0.25	5.00 ± 0.35	6.70 ± 0.46
***Bacillus subtilis***	1.50 ± 0.10	2.60 ± 0.18	3.90 ± 0.27	5.10 ± 0.35	6.90 ± 0.48
***Klebsiella pneumonia***	0.80 ± 0.05	2.10 ± 0.14	3.20 ± 0.22	4.50 ± 0.31	6.60 ± 0.46
***Staphylococcus aureus***	1.70 ± 0.11	2.80 ± 0.19	4.20 ± 0.29	5.50 ± 0.38	7.40 ± 0.51

## Data Availability

Data are contained within the article.

## Supplementary Materials

The following supporting information can be downloaded at: https://www.mdpi.com/article/10.3390/md23060224/s1, Table S1. Qualitative compound reported in crude extract; Table S2. Qualitative compounds reported in DBE Fraction 1; Table S3. Qualitative compound reported in DBE Fraction 2. Ref. [73] is cited in the Supplementary Materials.
