# Supplementary material for: Exploration of Bioactive Compounds, Antioxidant and Antibacterial Properties, and Their Potential Efficacy Against HT29 Cell Lines in Dictyota bartayresiana
Source: Mar Drugs. 2025 May 23;23(6):224. doi: 10.3390/md23060224 (PMC12194281; doi:10.3390/md23060224)
Supplement: Supplementary file 1 [file marinedrugs-23-00224-s001.zip › marinedrugs-3542595-supplementary.pdf]

Exploration of bioactive compounds, antioxidant and antibacterial properties, and their potential efficacy against HT29 cell lines in *Dictyota bartayresiana*

D. Swarna Bharathi [1,*], A. Boopathy Raja [1], Suganthi Nachimuthu [2], S. Thangavel [3], Karthik Kannan [4], S. Shanmugan [5] and Vinaya Tari [6,*]

[1]PG and Research Department of Zoology Nehru Memorial College (Affiliated to Bharathidasan University) Puthanampatti- 621004.Tamil Nadu, India. swarnaraj93@gmail.com

[2]PG and Research Department of Physics, Government Arts College (Autonomous) (Affiliated to Bharathidasan University), Karur– 639 005, Tamil Nadu, India. suganthiphyupm@gmail.com

[3]Department of Physics, C.S.I. Bishop Solomon Doraisawmy College of Arts and Science, Karur – 639 001, Tamil Nadu, India. suhithangam@gmail.com

[4]Department of Mechanical Engineering, Advanced Institute of Manufacturing with High-Tech Innovations, National Chung Cheng University, Chia-Yi, 621301, Taiwan. karthikk@ccu.edu.tw

[5]Research Centre for Solar Energy, Integrated Research and Discovery, Department of Physics, Koneru Lakshmaiah Education Foundation, Green Fields, Vaddeswaram, Guntur 522502, Andhra Pradesh, India

[6]Department of Biology, Faculty of Science and Technology, Universitas Airlangga, Surabaya, East Java, 60115, Indonesia.

*Correspondence: swarnaraj93@gmail.com (D.S.B.); drvinaya89@outlook.com (V.T.)

Table S1 Qualitative compound reported in crude extract

Compound Label	RT	Name	DB Formula	Hits (DB)	Biological activity
Cpd 1: Benzene, 1,3-dimethyl-	3.131	Benzene, 1,3-dimethyl-	C_8H_{10}	10	Not reported
Cpd 7: Cyclotetrasiloxane, octamethyl-	3.553	Cyclotetrasiloxane, octamethyl-	$C_8H_{24}O_4Si_4$	3	Not reported
Cpd 27: Ethyl (S)-(+)-mandelate	5.113	Ethyl (S)-(+)-mandelate	$C_{10}H_{12}O_3$	3	Antidiabetic activity [1]

Cpd 28: Benzoic acid, ethyl ester	5.32	Benzoic acid, ethyl ester	$C_9H_{10}O_2$	6	Food additive [1]
Cpd 31: Naphthalene	5.632	Naphthalene	$C_{10}H_8$	10	Not reported
Cpd 33: 1,2-Benzisothiazole	6.128	1,2-Benzisothiazole	C_7H_5NS	7	Not reported
Cpd 54: Quinoline, 1,2-dihydro-2,2,4-trimethyl-	9.524	Quinoline, 1,2-dihydro-2,2,4-trimethyl-	$C_{12}H_{15}N$	2	Antioxidant, antibacterial and anticancer activity [2,3]
Cpd 55: 1H-Indole, 2-(1,1-dimethylethyl)-	9.782	1H-Indole, 2-(1,1-dimethylethyl)-	$C_{12}H_{15}N$	1	Not reported
Cpd 64: Benzoic acid, 4-ethoxy-, ethyl ester	11.027	Benzoic acid, 4-ethoxy-, ethyl ester	$C_{11}H_{14}O_3$	4	Antioxidant, anticancer activity and antibacterial, [2,4]
Cpd 72: Hexadecane	12.578	Hexadecane	$C_{16}H_{34}$	1	Antifungal activity [5]
Cpd 73: 1(2H)-Naphthalenone, 3,4-dihydro-3,3,6,8-tetramethyl-	12.703	1(2H)-Naphthalenone, 3,4-dihydro-3,3,6,8-tetramethyl-	$C_{14}H_{18}O$	2	Not reported
Cpd 74: Benzothiazole, 2-(methylthio)-	12.74	Benzothiazole, 2-(methylthio)-	$C_8H_7NS_2$	1	Not reported
Cpd 84: Hexadecane	14.631	Hexadecane	$C_{16}H_{34}$	2	Antifungal activity [5]
Cpd 89: 9H-Fluorene, 9-	16.371	9H-Fluorene, 9-methylene-	$C_{14}H_{10}$	10	Not reported

methylene-					
Cpd 90: Hexadecane	16.677	Hexadecane	$C_{16}H_{34}$	1	Antifungal activity [5]
Cpd 95: 1,2-Benzenedicarboxyli c acid, bis(2-methylpropyl) ester	17.921	1,2-Benzenedicarboxyli c acid, bis(2-methylpropyl) Ester	$C_{16}H_{22}O_4$	10	Not reported
Cpd 100: Hexadecanoic acid, methyl ester	19.715	Hexadecanoic acid, methyl ester	$C_{17}H_{34}O_2$	2	Anti-fungal and antibacterial [6]
Cpd 101: Benzothiazole, 2-(2-hydroxyethylthio)-	19.892	Benzothiazole, 2-(2-hydroxyethylthio)-	$C_9H_9NOS_2$	1	Not reported
Cpd 104: Dibutyl Phthalate	20.483	Dibutyl phthalate	$C_{16}H_{22}O_4$	10	Not reported
Cpd 120: Eicosyl Acetate	29.134	Eicosyl acetate	$C_{22}H_{44}O_2$	1	Not reported
Cpd 138: Phthalic acid, di(2-propylpentyl) ester	35.215	Phthalic acid, di(2-propylpentyl) ester	$C_{24}H_{38}O_4$	10	Not reported

Table S2 Qualitative compounds reported in DBE Fraction 1

**Compound Label**	**RT**	**Name**	**DB Formula**	**Hits (DB)**	**Biological activity**
Cpd 1: Benzene, 1,3-dimethyl-	3.132	Benzene, 1,3-dimethyl-	C_8H_{10}	10	Not reported

Cpd 7: Cyclotetrasiloxane, octamethyl-	3.553	Cyclotetrasiloxane, octamethyl-	$C_8H_{24}O_4Si_4$	3	Anticancer activity [7]
Cpd 29: Benzoic acid, ethyl ester	5.325	Benzoic acid, ethyl ester	$C_9H_{10}O_2$	4	Food additive [1]
Cpd 32: Naphthalene	5.631	Naphthalene	$C_{10}H_8$	10	Not reported
Cpd 56: Bicyclo[5.2.0]nonane, 2-methylene-4,8,8-trimethyl-4-vinyl-	9.245	Bicyclo[5.2.0]nonane, 2-methylene-4,8,8-trimethyl-4-vinyl-	$C_{15}H_{24}$	4	Not reported
Cpd 68: Benzoic acid, 4-ethoxy-, ethyl ester	11.029	Benzoic acid, 4-ethoxy-, ethyl ester	$C_{11}H_{14}O_3$	4	Antioxidant, anticancer activity and antibacterial, [2,4]
Cpd 74: Diethyl Phthalate	12.287	Diethyl Phthalate	$C_{12}H_{14}O_4$	10	Not reported
Cpd 76: Hexadecane	12.576	Hexadecane	$C_{16}H_{34}$	1	Antifungal activity [5]
Cpd 98: Phthalic acid, hept-4-yl isobutyl ester	17.915	Phthalic acid, hept-4-yl isobutyl ester	$C_{19}H_{28}O_4$	10	Not reported
Cpd 108: Phthalic acid, butyl hept-4-yl ester	20.485	Phthalic acid, butyl hept-4-yl ester	$C_{19}H_{28}O_4$	10	Not reported
Cpd 134: Phthalic acid, di(2-propylpentyl) ester	35.213	Phthalic acid, di(2-propylpentyl) ester	$C_{24}H_{38}O_4$	10	Not reported
Cpd 143: 1,4-		1,4-Benzenedicarboxyli c			

Benzenedicarboxyli c acid, bis(2-ethylhexyl) ester	37.365	acid, bis(2-ethylhexyl) Ester	$C_{24}H_{38}O_4$	2	Not reported

Table S3 Qualitative compound reported in DBE Fraction 2

Compound Label	RT	Name	DB Formula	Hits (DB)	Biological activity
Cpd 4: Phenol, 2-methyl-	4.127	Phenol, 2-methyl-	C_7H_8O	10	Not reported
Cpd 5: Naphthalene	5.636	Naphthalene	$C_{10}H_8$	10	Not reported
Cpd 6: p-Isopropenylphenol	6.958	p-Isopropenylphenol	$C_9H_{10}O$	10	Not reported
Cpd 7: Quinoline, 1,2-dihydro-2,2,4-trimethyl-	9.52	Quinoline, 1,2-dihydro-2,2,4-trimethyl-	$C_{12}H_{15}N$	2	Antioxidant, antibacterial and anticancer activity [2,3]
Cpd 9: Pentanoic acid, 5-hydroxy-, 2,4-di-t-butylphenyl esters	10.65	Pentanoic acid, 5-hydroxy-, 2,4-di-t-butylphenyl Esters	$C_{19}H_{30}O_3$	2	Not reported
Cpd 10: Benzoic acid, 4-ethoxy-, ethyl ester	11.035	Benzoic acid, 4-ethoxy-, ethyl ester	$C_{11}H_{14}O_3$	4	Antioxidant, anticancer activity and antibacterial, [2,4]
Cpd 11: n-Tridecan-1-ol	12.418	n-Tridecan-1-ol	$C_{13}H_{28}O$	10	Not reported

Cpd 15: n-Pentadecanol	16.527	n-Pentadecanol	$C_{15}H_{32}O$	10	Antibacterial activity [2]
Cpd 19: n-Nonadecanol-1	21.831	n-Nonadecanol-1	$C_{19}H_{40}O$	10	Not reported
Cpd 23: Acetic acid n-octadecyl Ester	29.13	Acetic acid n-octadecyl ester	$C_{20}H_{40}O_2$	3	Not reported

References

[1] Christopher I, Keeling, Keith N Slessor, Heather A. Higo, and Mark L Winston (2003) New components of the honey bee (Apis mellifera L.) queen retinue pheromone. 4486–4491 PNAS vol. 100 no. 8. doi10.1073pnas.0836984100.

[2] Manjunatha JR, Bettadaiah BK, Negi PS, Srinivas P (2013) Synthesis of quinoline derivatives of tetrahydrocurcumin and zingerone and evaluation of their antioxidant and antibacterial attributes. Food Chemistry 136, 650-58.

[3] Kazue Takahata , Hiroshi Katsuki , Yutaka Kobayashi , Shizuko Muraoka , Fumio Yoneda , Toshiaki Kume , Satoshi Kashii , Yoshihito Honda , Akinori Akaike (2003) Protective effects of selegiline and desmethylselegiline against N-methyl-D-aspartate-induced rat retinal damage. European Journal of Pharmacology 458, 81 – 89.

[4] Guo-qiang Zheng, Patrick M, Kenney, Jilun Zhang and Luke KT, Lam (1992) Inhibition of benzo[a]pyrene-induced tumorigenesis by myristicin, a volatile aroma constituent of parsley leaf oil. Carcinogenesis vol.13 no.10 pp.1921-1923.

[5] Ilaine TS Gehrke, Alexandre T Neto, Marcelo Pedroso, Clarice P Mostardeiro, Ivana BM Da Cruz, Ubiratan F Silva, Vinicius Ilha, Ionara I Dalcol, Ademir F Morel (2013) Antimicrobial activity of Schinus lentiscifolius (Anacardiaceae). Journal of Ethnopharmacology 148, 486–491.

[6] Chandrasekaran M, Senthilkumar A. and Venkatesalu V. (2011). Antibacterial and antifungal efficacy of fatty acid methyl esters from leaves of *Sesuvium portulacastrum* L. *European Review for Medical and Pharmacological Sciences*, 15, 775-780.

[7] Xue Xiao, Juan Zou, Yin Fang, Yibo Meng, Chao Xiao, Jiaxin Fu, Shiyu Liu, Peng Bai and Yuan Yao

(2018) Fisetin and polymeric micelles encapsulating fisetin exhibit potent cytotoxic effects towards ovarian cancer cells. BMC Complementary and Alternative Medicine 18:91.
